# California State Veterinary Medical Association

**Published:** 1894-01

**Authors:** R. A. Archibald

**Affiliations:** Secretary; Sacramento, Cal.


### CALIFORNIA STATE VETERINARY MEDICAL ASSOCIATION.

The California State Veterinary Medical Association held its annual meeting at the State House Hotel, Sacramento, Cal., Dec. 13th. 1893. Owing to the absence of the President the meeting was called to order by the Vice-President, Dr. H. F. Spencer.

Upon roll call the following gentlemen responded to their names, viz.: Drs. Maclay, McCollum, Spencer, Sr., Spencer, Jr., Orvis, Fox, Pierce, Williams, Hogarty and Archibald.

The minutes of the previous meeting were read and approved.

The Treasurer's report was then read. The report showed the Association to be in a rather straitened condition financially, owing to the large amount of money paid out in the past year on expenses incurred in legislative matters, etc. The report also showed that there was considerable money standing out, due the Association by members who are in arrears. On motion by Dr. Fox, the report was accepted.

The Secretary's report was then read. The report showed the Association to be in a flourishing condition numerically, and the roll call showed a large increase. The Secretary stated that he had had about a dozen applications for membership in the past month. He reviewed the work done by him in the past year; he also reviewed in detail the work done in legislative matters by the Association in the past year; also what the Association intended to do in the future. He complained of the stand taken by the majority of the members of the Association; he also complained of the lack of assistance he had received; also the lack of interest taken by the members in making the meetings interesting and instructive; he intimated that Association matters were not matters of dollars and cents or individual benefit, but were for the advancement of the veterinary profession and the protection of the public in the State of California. On motion of Dr. Orvis, the report was accepted.

The Board of Examiners reported favorably on the names of applicants for membership referred to them at last meeting, with the exception of I. B. Dalziel, of San Francisco. The Board said that this applicant was a veterinary dentist, and had not received a license from the State Board of Examiners, therefore was not eligible for membership. On motion of Dr. Fox, the Board's report was accepted, and upon motion by Dr. Pierce, the names of the applicants, as recommended by the Board of Examiners, were elected as active members of the Association.

The names of those elected are as follows, viz.: Dr. H. Lemker, Bakersfield; Dr. A. Paterson, San Francisco; Dr. G. F. Faulkner, Salinas; Dr. A. Bobins, San Francisco; Dr. H. Fabbi, San Francisco; Dr. H. A. Forest, Santa Cruz; Dr. A. S. Williams, Marysville; Dr. H. R. Jackson and J. J. Hogarty, Oakland, and Dr. J. H. Eddy, Stockton.

The Board of Directors reported favorably and recommended the adoption of the proposed amendments to the Constitution and By-Laws referred to them at last meeting

On motion of *Dr. Maclay*, the Board's report was accepted and the amendments as recommended by the Board were adopted.

On motion of Dr. Maclay, the Secretary was instructed to procure printed copies of the Constitution and By-Laws as amended, and that he be empowered to draw on the Treasurer for the amount necessary for same.

The Secretary then read a communication from Dr. R. H. Power, of Stockton, in which he stated that he desired to withdraw from the Association. On motion by *Dr. Fox*, Dr. Power's resignation was accepted.

Several communications from absent members were read by the Secretary expressing regrets for being unable to attend the meeting.

The following bills were allowed and ordered paid: J. J. Evans, $15.00, for printing certificates; R. A. Archibald, $23.35, for expenses incurred by him as Secretary during the past year.

The Chair declared that nominations for officers for the ensuing year were in order, because at last meeting it was impossible to find a member who was willing to accept the office of Secretary. *Dr. Maclay* nominated *Dr. R. A. Archibald* for Secretary, whereupon on motion of *Dr. Orvis*, the nominations were closed.

The Secretary was requested to read the result of the nominations, which he did as follows :

*President*—Dr. H. A. Spencer, San Jose.

*Vice-President*—Dr. Ward B. Rowland, Pasadena

*Secretary*—Dr. R. A. Archibald, Sacramento.

*Treasurer*—Dr. D. F. Fox, Sacramento.

*Board of Examiners*—Drs. Maclay, Orvis, Egan, Rowland and Whittlesey.

*Board of Directors*—The several officers of the Association,

*Dr. Maclay* said that as there was but one nomination for each office, he moved that those nominated for the several offices be elected by acclamation. The motion was duly seconded by Dr. Pierce and carried.

*Dr. Fox* moved, seconded by Dr. Orvis, that the newly-elected President take his seat. Carried.

The newly-elected President upon taking his seat made a few appropriate and well chosen remarks. He thanked the members for the great honor they had done him and promised to do all in his power to make the meetings interesting and instructive, and in that way further the interests of the Association. He deplored the lack of attendance to the meetings and said he would leave no stone unturned to bring the members of the veterinary profession in the State of California together and have them work hand in hand for the advancement of the profession in this great and glorious State.

Under the head of Reading of Papers, etc., and discussions, the Secretary was called upon to read a few notes he had prepared, his subject being "The Excision of the Thyro-arytenoidean Ligament and a Portion of the Arytenoid Cartilage for Roaring."

Before reading his essay, the Essayist extended a cordial invitation to the members to witness the operation of Laryngotomy as performed by him at the Sacramento Veterinary Hospital at 9 A. M. next day.

The Essayist went on to describe his method of performing the operation ; he also noticed the chief differences between Prof. Fleming's operation and the operation as performed by him.

The paper was followed by an interesting discussion.

*Dr. Orvis* criticised the operation as performed by the Essayist in every detail. He said he was unwilling at that time to admit that the Essayist's method was preferable to the Fleming method ; he said he had performed the Fleming operation and had failed to find any of the bad results mentioned by the Essayist follow the operation; at the same time, owing to lack of experience in this matter, he was not prepared to condemn the operation as performed by the Essayist.

*Dr. Maclay* stated that the subject of Laryngotomy was one in which he had not had much experience, but he agreed with the Essayist in that Fleming's method had not been successful. He complimented the Essayist very highly on the originality of his remarks ; he said he believed that the matter could be more thoroughly discussed after they had witnessed the Essayist perform the operation.

*The Essayist* in defending his paper gave his reasons for believing his method of operating to be superior to the Fleming method. He produced a dried larynx of a horse and pointed out on the specimen where he believed Prof. Fleming erred when he performed the operation.

*Dr. Orvis* then arose and said, that if the operation, as performed by the essayist, was followed by beneficial results, he was willing to admit that it was superior to Fleming's method. He said this was a subject in which he was very much interested, and that he would like to have it discussed by every member present.

*Dr. Maclay* said, that as no one present knew much about this operation it was useless to discuss it.

*Dr. Orvis* desired to know how we were to learn anything about this operation unless we discuss it, as it was very seldom we could procure a roarer to operate on ; also that we have to rely on these discussions to gain knowledge on this subject, as there was no literature on it.

After the subject matter had been discussed by most of the members present, he President closed with a few remarks. He agreed with Dr. Maclay, in that the subject could be discussed more intelligently after they had witnessed the operation. He said the essayist was to be commended for the originality of his revision of this operation, inasmuch as he has given us food for study and reflection, and which, if we determine to be the best mode of procedure, certainly reduces the magnitude of the operation, and simplifies it, also unquestionably makes recovery more rapid.

*Dr. Maclay* moved that the meeting adjourn to meet next day at 3 P. M. The motion was seconded by Dr. Fox and carried.

*Second Day's Session.*

On December 14th, at 9 A. M., the members of the California State Veterinary Medical Association assembled at the Sacramento Veterinary Hospital to witness the operation of laryngotomy, demonstrated by Dr. R. A. Archibald. He performed the operation after the method described by him in his essay. The members were unanimously pleased with his manner of operating, and all expressed a conviction that if the operation was followed by beneficial results, it was a superior method of operating than that described by Fleming.

The members then witnessed Dr. Orvis perform the operation of ovariotomy on a bitch. The members complimented the operator on the skilful manner in which he performed the operation.

The meeting of the California State Veterinary Medical Association convened December 14th at 3 P. M. The President, *Dr. H. A. Spencer*, called the meeting to order.

Business was resumed by the reading of an interesting paper on Glanders by *Dr. C. B. Orvis.* He gave a description of the disease, and cited a number of cases which he believed had recovered from this fatal malady. He also advanced the idea that one attack of glanders rendered an immunity from a second attack ; he also stated that it was his belief that horses who apparently recovered and did not show any symptoms of the disease for one year, had fully recovered, and he would pronounce such a horse sound. He closed with the reading of an item from the *American Veterinary Review*, which gave an account of a spontaneous recovery in a horse, written by a Russian veterinarian.

*Dr. McCollum* said he did not believe glanders could be cured, and he cited a number of cases in support of his belief. He said in those so called cases of spontaneous recovery the germ lies latent in the animal's system, and will develop when the animal is exposed to hardships. He cited an instance of an animal who was supposed to have recovered, and who changed hands a great many times and finally, after a lapse of five years, died from acute glanders. He and a brother practitioner visited the different places the animal had been kept, and they found that said case was the cause of seventeen deaths among horses.

*The Secretary* said that although he was unwilling at the present time to say that glanders could be cured, he was of the opinion that the few cases that would succumb to treatment would not compensate for the risk run in letting these so-called cases of spontaneous recovery run at large. He believed these cases to be more dangerous to a community than cases which showed more aggravated symptoms, as they were less liable to cause suspicion. He cited a number of cases to show that these cases were very dangerous to any community. He said this was a matter which would be advisable to keep from the public, as there were a great many ignorant people, who would take advantage of the knowledge of the fact that horses affected with glanders may recover and make it very unpleasant for those whose duty it was to condemn and destroy these cases.

*Dr. Maclay* said the time was come to have some one appointed to look after these matters, this person to be paid a salary sufficiently large so he could afford to do his duty. It was impossible for us to do our duty the way these matters are arranged at the present time In support of this statement he cited an instance, supposing a member of this Association is passing down the street and sees a horse affected with glanders tied to a hitching post. His duty as a man and citizen in that case would be to have the owner of the animal arrested. But what would be the consequence? He would lose the custom and friendship of that man and all his friends. The time is come to appoint one who could afford to make enemies in such cases.

*The Essayist* said he did not want to advance the theory that glanders was curable, but he mentioned these cases in order to provoke a discussion.

*The Secretary* asked the essayist if he had ever used mallein in these supposed recovered cases. He believed that if these cases that had been affected with glanders were subjected to a test with mallein they would develop the disease. He cited a number of cases in support of his idea.

*The Essayist* said he had never used mallein on these cases.

*Dr. McCollum* said that he believed that the use of mallein was invaluable in the diagnosis of glanders, especially in these mild cases.

*Dr. Fox* mentioned a case of glanders in a human being. A man in Monterey County, who refused to destroy some horses condemned by him, contracted the disease and died He said he again examined the stock belonging to this man, and found a great number had contracted the disease since his former examination.

*The Secretary* also cited a number of cases in which human beings had contracted the disease. He said it was his belief that a great many people died from glanders that we never heard of, because there were so many physicians who would not recognize the disease when they saw it

*Dr. Pierce* related a case in which he showed how a single case may spread the disease in a community. He said gypsies and horse traders were the means of carrying diseases from place to place.

*The President* closed the discussion by relating a case of a black horse which was brought to his hospital showing a very mild case of glanders He used mallein and sent the animal home. Next day he visited the horse and found him nearly dead. The owner told him that the horse had been suffering terribly since he brought him back from the hospital, and that he had rigors every hour or so.

He also mentioned a case where he had condemned twenty-one horses on a ranch belonging to Mr. D. Murphy. Six months later he again examined the stock and found seven cases. After both examinations he had the premises thoroughly disinfected, the fences, barns, etc., were whitewashed. The disease confined itself to the working horses, and the horses running in the pasture had escaped the ravages of the disease. The cause of this he believed was that the germ of the disease was distributed through the barns and stables only.

On motion of Dr. Fox the meeting adjourned to meet that evening at 7.30 P. M.

The meeting of the California State Veterinary Medical Association convened at 7.30 P. M.

The meeting was called to order by the President, Dr. H. A. Spencer.

*The President* then called upon Dr. Maclay to entertain the meeting with a paper

*Dr. Maclay* arose and said that his paper was entitled "The Relation of Animal Diseases to the Public Health."

Before reading his essay he stated that he had tried to make his paper as concise as possible, but that it was impossible to deal with the subject without writing an exhaustive treatise. He warned the members that it would take him about an hour to read his paper, and that if any of them were tired they had better leave before he began to read.

He went on to read his paper, which proved to be one of the most interesting and instructive papers that had ever been read before the Association. He mentioned the following diseases, viz.: Trichiniasis, Hog Cholera, Tænia Mediocanellata, Eczema, Epizootica, Tuberculosis, Anthrax, Anthracoid Diseases, Glanders, etc., giving a description of each disease, also detailing the different methods of preventing and eradicating them. He then went on and described the proper method of regulating sanitation, and protecting the public from the ravages of these diseases in the State. He reviewed the laws pertaining to these matters, pointing out where the laws were inadequate to control these matters. He said the Governor should be empowered to select a commission of veterinarians and lawyers to draft proper laws and regulations for the purpose of suppressing contagious diseases. A State veterinarian or inspector general should be appointed to look after these matters and enforce laws passed by the Legislature. The position should not, as is generally the case, be filled by those whose only recommendation is a political pull. It was true some of these diseases do not exist in the State at the present time, yet we never know when they may be introduced here, and that we should be prepared for them when they do come. He reviewed the condition of San Francisco, Sacramento, Oakland, Los Angeles and other large cities from a sanitary standpoint. He cited numerous instances where these diseases had been transmitted to the human family. He closed with a few remarks on the injurious effects to be got from eating the flesh of animals which had been killed while suffering from sporadic diseases such as Actinomycosis, Puerperal Fever, etc.

*The President* opened the discussion with a few well chosen remarks, he complimented the essayist very highly on the able manner in which he had treated this matter. He said it was a cause of grief that a paper so comprehensive, and so carefully prepared, should have been listened to by so few. He wished the matter could be laid before every legislator. He was glad of the privilege of listening to the paper. He hoped the day would come when the matter would be taken up by the press and public, and that they insist that proper legislation be enacted to regulate the sale of diseased meat and milk. We must expect bitter opposition as there were so many of lax morals interested in making a profit in the sale of diseased meat and milk.

*Dr. McCollum* said this was only one of a number of splendid and exhaustive papers on various subjects that had been read before the Association. It was a magnificent production, and one on which the public should have its eyes opened. He had been at the slaughter houses and seen diseased animals slaughtered and hung up beside healthy carcasses, and he had seen calves from diseased cows killed, hung up, and sold in the market; also sheep whose bodies were covered with scabs. Yet he said, that if a member of this Association was to go to his local Board of Health, or County Supervisors, and ask them to appoint a competent man to look after these matters, they would imagine he was looking for a job, and he would get no credit or satisfaction for asking it for the sake of his fellow-beings.

*The Secretary* said he felt it his duty to compliment the essayist on the able and masterly manner in which he had treated on this subject, he also thanked the essayist for the pleasure he had received in listening to his paper. He intended before the meeting adjourned to move that the Association instruct the secretary to have copies of the paper printed, and a copy be sent to each and every member of this Association, also a copy be sent to the members of the State Board of Health and local Boards of Health. On the subject of tuberculosis he said, that from what he had read and what little experience he had had on these matters, he had come to the conclusion that tuberculosis was a disease peculiar to the bovine, and that the germ of tuberculosis had greater difficulty in maintaining itself in the human family than in the bovine.

In support of his theory he cited instances. Certain countries where the inhabitants did not use the meat and milk of cattle tuberculosis was an unknown disease, but after the introduction of cattle into these countries, the disease became common among the natives. He believed that civilization was the greatest propagator of disease known to him. You often see a healthy man and wife, with three or four children. one or two of these children are puny little beings, while the others are strong, healthy and robust. If you investigate the matter you will find, in every instance, that at the time the mother gave birth to these puny children she was so circumstantiated that she was unable to nurse the children, but brought them up on cow's milk, and it generally happened that she secured the milk from a Jersey cow, of which breed of cows he believed that 50 per cent. were affected with tuberculosis. He also believed that 25 per cent. of the dairy cattle in this State were affected with tuberculosis.

He mentioned a case where there were twenty horses affeeted with glanders dumped into the Sacramento River a short time ago, this being the source from which the people of Sacramento obtained their drinking water. He mentioned a case of anthrax he had seen a short time ago in which a horse had contracted the disease from drinking water from a stream that flowed through a slough, in which some cattle which had died from anthrax had been dumped some time before.

*Dr. Fox* cited a number of cases of glanders he had investigated when he was County Inspector for Monterey County.

*Dr. Spencer, Jr.*, mentioned a case of glanders in a man.

*Dr. McCollum* mentioned a case where he saw a man suffering from trichinosis. He said it was one of the most loathsome sights he had ever witnessed.

There was considerable more discussion of this subject, all tending to the same point. That there should be some steps taken to prevent the transmission of these diseases to the human family.

Under the head of new business the Secretary moved, seconded by Dr. Fox, that the Secretary be instructed to have copies of Dr. Maclay's paper printed and that a copy of the same be sent to the members of the State Board of Health and local Boards of Health, also to every member of the Association, and that an assessment of one dollar be levied on each and every member of the Association to defray the expense of printing same.

The motion was carried.

*The President* appointed the following named gentlemen to prepare essays and read them at the next meeting, viz.: Drs. Fox, Spencer, Jr., Whittlesey and Pieree. In connection with this the President made a few remarks. He said that when he appointed a member to read an essay, he expected him to do so, and he intended that he should read a paper.

On motion by Dr. Fox a vote of thanks was tendered the essayists for the able and masterly manner in which they had entertained the meeting.

There being no further business before the meeting, it adjourned to meet in San Francisco, March 11th, 1894, at the Baldwin Hotel.

R. A. ARCHIBALD, *Secretary.*

SACRAMENTO, Cal.

